# *Pneumocystis jirovecii* Pneumonia in Non-HIV Patients Recovering from COVID-19: A Single-Center Experience

**DOI:** 10.3390/ijerph182111399

**Published:** 2021-10-29

**Authors:** Ivan Gentile, Giulio Viceconte, Amedeo Lanzardo, Irene Zotta, Emanuela Zappulo, Biagio Pinchera, Riccardo Scotto, Nicola Schiano Moriello, Maria Foggia, Agnese Giaccone, Gaetana Messina, Paola Salvatore, Antonio Riccardo Buonomo, Federico II COVID-19 Team

**Affiliations:** 1 Department of Clinical Medicine and Surgery, Section of Infectious Disease, University of Naples “Federico II”, 80131 Naples, Italy; ivan.gentile@gmail.com (I.G.); a.lanzardo@gmail.com (A.L.); ire.zotta@gmail.com (I.Z.); e.zappulo@gmail.com (E.Z.); biapin89@virgilio.it (B.P.); ri.scotto@gmail.com (R.S.); veghan@gmail.com (N.S.M.); mariafoggia@alice.it (M.F.); agnesegiaccone94@gmail.com (A.G.); antonioriccardobuonomo@gmail.com (A.R.B.); 2 Toracic Surgery Unit, University of Campania “Luigi Vanvitelli”, 80138 Naples, Italy; adamessina@virgilio.it; 3 Department of Molecular Medicine and Medical Biotechnology, University of Naples “Federico II”, 80131 Naples, Italy; psalvato@unina.it

**Keywords:** COVID-19, *Pneumocystis jirovecii*, invasive fungal infections, corticosteroids, lymphopenia

## Abstract

Objective: to describe a single-center experience of *Pneumocystis jirovecii* pneumonia (PJP) in non-HIV patients recovering from COVID-19. Methods: We report the cases of five non-HIV patients with COVID-19 who also developed PJP at a University Hospital. Results: With the exception of one subject, who experienced an atypical and prolonged course of COVID-19, all the patients developed PJP after the clinical resolution of COVID-19 pneumonia. All but one patient had no pre-existing immunosuppressive conditions or other risk factors for PJP development at COVID-19 diagnosis. Nonetheless, following the course of COVID-19 infection, all the patients fulfilled at least one host factor for PJP; indeed, all the patients had received at least 2 weeks of high-dose steroids and three out of five had a CD4+ cell count <200/mm^3^. Conclusions: The use of corticosteroids for COVID-19 respiratory impairment seems to be the most common risk factor for PJP, together with viral-induced and iatrogenic lymphopenia. The worsening in respiratory function and the characteristic radiological picture during or after COVID-19 pneumonia should raise the suspicion of PJP, even in immunocompetent patients. PJP primary chemoprophylaxis can be considered in selected high-risk COVID-19 patients, but further studies are needed.

## 1. Background

Coronavirus Infectious Disease 19 (COVID-19) is a disease caused by Severe Acute Respiratory Syndrome Coronavirus 2 (SARS-CoV-2), causing severe interstitial pneumonia associated with respiratory failure [1] Similarly to severe influenza, invasive fungal infections are not uncommon in COVID-19 patients, with invasive pulmonary aspergillosis more frequently reported [2].

Although respiratory tract colonization with *Pneumocystis jirovecii* reaches 9% in COVID-19 patients admitted to intensive care units, only a few cases of *Pneumocystis jirovecii* pneumonia (PJP) have been reported so far, almost exclusively in individuals with concurrent immunosuppression (HIV, treatment for autoimmune diseases, solid organ transplantation) [2,3,4]. In this study, we describe a case series from a single-center experience of five patients who received a diagnosis of PJP, of which one had classical risk factors for PJP and the others were immunocompetent before COVID-19 onset. The clinical features and risk factors for PJP of our patients are presented in Table 1.

## 2. Patients and Methods

The described cases were observed in the COVID-19 Infectious Disease Unit of Federico II University Hospital: a non-ICU ward that has managed around 450 cases of moderate-to-severe COVID-19 since March 2020.

The diagnosis of PJP was considered ‘proven’ if P. jirovecii was detected on bronchoalveolar lavage fluid (BALF) with immunofluorescence assay (IFA), ‘probable’ in the presence of host factors, clinical features and mycrobiological evidence (PCR for P. jirovecii on respiratory samples or serum Beta-D-Glucan assay), and ‘possible’ in the absence of mycrobiological evidence, according to the European Organization for Research and Treatment of Cancer and the Mycoses Study Group (EORTC/MSGERC) criteria [5]. BALF was collected bed-side by an expert bronchoscopist (GM) using CT-scan to guide sampling and was sent to the laboratory within 1 h. BALF was processed with MONOFLUO™ KIT (Axis-Shield Diagnostics Limited) for IFA and observed by an expert microbiologist (PS). The test was considered positive in the case of visualization of five or more fluorescent oocysts over the whole slide.

Written informed consent was obtained from the patients before performing the bronchoscopy and for the publication of their clinical data and radiological pictures as well.

## 3. Case Series

### 3.1. Patient 1

A description of the first case has already been published as a single case report, in which we presented a confirmed case of PJP (*P. jirovecii* direct immunofluorescence tested positive on BALF) in an immunocompetent patient 40 days after COVID-19 symptom onset. The patient was admitted for severe COVID-19 and treated with CPAP, low molecular weight heparin, ceftaroline, and 6 mg of dexamethasone for 10 days with tapering and discharged 18 days later in an improved condition. He was then readmitted for fever, cough and respiratory failure 18 days after discharge. He underwent a bronchoscopy with BALF collection, which was positive for *P. jirovecii* IFA. The patient experienced a full clinical and radiological recovery from PJP after a 21 day course of trimethoprim-sulfamethoxazole (CTX) and steroidal therapy [6].

### 3.2. Patient 2

**Figure 1:**
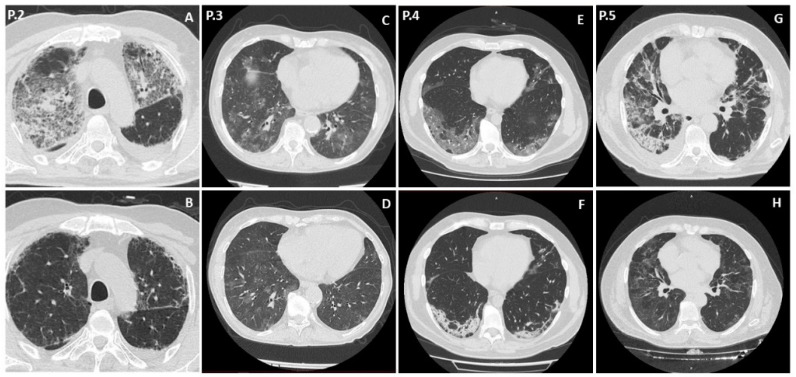
Radiological characteristics of the patients. Patient 2 (P.2): (**A**) HRCT at PJP diagnosis; (**B**) HRCT after CTX therapy, 21 days after PJP diagnosis. Patient 3 (P.3): (**C**) HRCT at PJP diagnosis; (**D**) HRCT after CTX therapy, 30 days after PJP diagnosis. Patient 4 (P.4): (**E**) HRCT at PJP diagnosis; (**F**) HRCT after CTX therapy, 20 days after PJP diagnosis. Patient 5 (P.5): (**G**) HRCT at PJP diagnosis; (**H**) HRCT after CTX therapy, 26 days after PJP diagnosis. HRCT = high-resolution CT-scan; PJP = Pneumocystis jirovecii pneumonia; CTX = trimethoprim-sulfamethoxazole.

A 68 year-old man was admitted to the intensive care unit (ICU) for severe acute respiratory failure ten days after COVID-19 symptom onset. His past medical history was unremarkable. He was treated at home with prednisone 75 mg daily from symptom onset to ICU admission. Upon ICU admission, he was placed on noninvasive ventilation (NIV) for 10 days and received enoxaparin 6000 UI and dexamethasone 6 mg intravenously (i.v.) with progressive tapering. A chest X-ray performed upon ICU admission revealed bilateral ground glass opacities (GGO) and bilateral pleural effusion. Fifteen days after ICU admission, the patient was moved to the Infectious Disease Unit (IDU), with a SpO2 of 98% on nasal cannula 2 L/min, no corticosteroid therapy and negative nasopharyngeal swab (NFS) for SARS-CoV-2. Upon IDU admission he had a white blood cell count (WBC) of 7500/mm^3^, an absolute lymphocyte count (ALC) 560/mm^3^, C-reactive protein (CRP) 1.2 mg/dL, and normal LDH. Eight days later, fever and dyspnea appeared. His CRP was 29 mg/dL, LDH 300 U/L, WBC 7410/mm^3^, ALC 590/mm^3^, with 24% CD4 (absolute count 141 cell/mm^3^), and 17% CD8. For this reason, we performed a high-resolution chest CT-scan (HRCT), which revealed peripheral and central GGO and bilateral pleural effusion (Figure 1A). The same day, he was moved again to the ICU to receive NIV. No bronchoscopy was performed due to the patient’s critical condition; nonetheless, PJP was suspected on the basis of the clinical and radiological picture. Therefore, therapy with CTX 20 mg/kg in 4 daily i.v. doses, methylprednisolone 40 mg BID i.v. was started. Three Beta-D-Glucan assays (BDG) performed during the hospitalization were negative. He was treated with NIV for five days and then moved again to the IDU in an improved respiratory condition. He received CTX and tapered corticosteroid therapy for 21 days. His condition progressively improved, with a SpO2 of 97% on nasal cannulae 2 L/minute thirty-two days after the first parenteral administration of CTX. Chest High-resolution Computer Tomography (HRCT) performed 50 days after admission showed an improvement in the patient’s parenchymal lesions (Figure 1B). 

### 3.3. Patient 3

A 63 year-old woman, affected by arterial hypertension and non-Hodgkin lymphoma treated with R-CHOP chemotherapy and currently under maintenance therapy with subcutaneous rituximab.

The patient experienced a relapsing pattern of COVID-19 symptoms for 3 months and she was hospitalized in a peripheral hospital for two months for COVID-19 mild pneumonia, for which she received steroidal, antibiotic and anticoagulant therapy. She underwent a first HRCT upon admission during her first hospitalization, which revealed an initial pattern of bilateral GGO. Another HRCT performed later demonstrated an improvement in the interstitial lesions, followed by a new worsening of both clinical and radiological pictures. She was then moved to our hospital.

Upon admission, she was in a good clinical condition, with an oxygen saturation of 93% on room-air and low-grade fever. Blood tests revealed an absolute lymphocyte count of 490/mm^3^, with 0% C19+, 19% CD4+ (absolute count 93/mm^3^) and 58% CD8+. Blood, urine and sputum cultures were negative, as were serological tests for intracellular bacteria and Legionella pneumophila, a molecular assay for respiratory viruses, and seriate BDG assays.

She received ceftaroline 600 mg BID and levofloxacine 750 mg QD for 6 days, with no improvement in her fever or her CRP levels.

On the 116th day of disease another HRCT was performed (Figure 1C), which revealed bilateral GGO with a severity score of 11/20 according to the Chung score [7]. A bronchoalveolar lavage fluid (BALF) was then collected two days later, on which *P. jirovecii* was detected using direct immunofluorescence. CTX 20 mg/kg p.o. daily, together with prednisone 20 mg BID, were started, with an improvement in blood gas exchanges and a reduction in CRP. Therapy with CTX was stopped after 21 days, with prednisone tapering. An HRCT performed at the end of the CTX therapy showed an improvement in the GGO, with a score of 6/20 (Figure 1D). After PJP resolution, the patient received a 10 days course of remdesivir in an attempt to clear SARS-CoV-2. This resulted in no benefits, so she received hyperimmune plasma for SARS-CoV-2. This was followed by a clinical improvement and repeated negativity for SARS-CoV-2 RNA on NFS 6 months after symptom onset.

### 3.4. Patient 4

A 55 year-old man, admitted to our Unit at the beginning of February 2021 for acute respiratory failure due to COVID-19 pneumonia. His past medical history was unremarkable. He reported the occurrence of fever 45 days before admission, when he performed a SARS-CoV-2 NFS that was positive. He started outpatient therapy with dexamethasone 4 mg QD for 5 days. His condition improved and a second NF swab 15 days after symptom onset tested negative for SARS-CoV-2. Fifteen days after the negative test, he was again febrile, with exertional dyspnea. A third NFS swab was performed and resulted positive. He started therapy with dexamethasone 4 mg BID, enoxaparin 4.000 IU BID, and azithromycin 500 mg QD, with no benefits. Ten days later, he was admitted to the IDU. On admission, the patient was febrile with a peripheral saturation of 88%. He had a WBC of 13,400/mm^3^, an ANC of 15,510/mm^3^, and an ALC of 240/mm^3^, with 26% CD4+ (absolute count 62/mm^3^) and 17% CD8+, CRP 32 mg/dL, procalcitonin 0.14 ng/mL, and LDH 338 U/L. He soon started oxygen therapy with a Venturi mask and empiric antimicrobial therapy with ceftobiprole 250 mg TID and levofloxacin 750 mg QD. A BDG test was performed, with a negative result, while SARS-CoV-2 was still detectable on NFS.

An HRCT performed on admission revealed a multiple GGO in all the lobes (Chung severity score 15/20), without consolidations (Figure 1E) [7]. Due to a lack of clinical response, a BALF was collected 3 days after admission, on which galactomannan antigen, CMV-DNA, VZV-DNA, PCR for respiratory viruses, microscopic and cultural examination for bacteria, fungi and mycobacteria and a GeneXpert test for Mycobacterium tuberculosis resulted negative, while *P. jirovecii* direct immunofluorescence tested positive. 

Therefore, we stopped the antimicrobial therapy and started treatment with CTX 20 mg/kg in 4 daily i.v doses and methylprednisolone 40 mg i.v. BID. This was associated with a rapid improvement in the patient’s respiratory symptoms and blood gas exchanges. Three weeks after admission, SARS-CoV-2 tested negative on NSF. A second HRCT scan was performed, which revealed a reduction in the previously described GGO (Chung severity score 7/20) [7] (Figure 1F). Fifty-five days after admission, the patient was in good clinical condition in room-air, with mild exertional dyspnea and low oxygen saturation while performing a 6 min walking test. 

### 3.5. Patient 5

A 75 year-old man with a history of chronic ischemic heart disease, persistent atrial fibrillation, type 2 diabetes mellitus. He started complaining of fever on the 1st of February 2020. Eight days later, he had a positive NFS for SARS-CoV-2 detection. He then started outpatient therapy with prednisone 25 mg daily and enoxaparin. Three days later, he was admitted to the IDU for worsening dyspnea. An HRCT on admission revealed bilateral GGO with a Chung score of 12/20 [7]. He was then treated with dexamethasone 6 mg i.v. daily for 10 days, enoxaparin 6000 UI QD, remdesivir for 5 days, and oxygen through a Venturi mask, and discharged 23 days later in an improved clinical condition and with a peripheral saturation of 96% in room-air, SARS-CoV-2 RNA still detectable on NFS, and a slightly increased CRP (1.3 mg/dL).

Three days after discharge, he started complaining of high-grade fever, asthenia, and exertional dyspnea (peripheral saturation 92% in room-air), and he was admitted again to our unit 2 days later. He had a negative NFS for SARS-CoV-2 RNA and a normal blood count, with ALC 2200/mm^3^, 46% CD4+ (absolute count 1012/mm^3^), 7% CD8+, LDH 280 U/L, and CRP 8.74 mg/dL. An HRCT revealed multiple GGO and bilateral consolidations (Figure 1G). On admission, BDG, C. pneumoniae and M. pneumoniae serology, and a urinary antigen of S. pneumoniae were negative. The day after admission, he underwent a bronchoscopy, which was complicated by bleeding from the upper airways, which affected the quality of the BALF sample. Galactomannan antigen, CMV-DNA, VZV-DNA, and PCR for respiratory viruses including SARS-CoV2, a GeneXpert test for Mycobacterium tuberculosis, and a *P. jirovecii* direct immunofluorescence were negative on BALF, while *Enterobacter cloacae*, *Acinetobacter baumanii*, and *Candida albicans* grew on cultures with <10^4 colony-forming units, but they were considered as colonizing. Nonetheless, due to suggestive radiological imaging, an empiric therapy for *P. jirovecii* with CTX 20 mg/kg in four daily i.v. doses and methylprednisolone 40 mg i.v. BID was started, with a rapid improvement in the respiratory picture and a progressive reduction in CRP. He was moved 15 days after admission to a respiratory rehabilitation unit in a good clinical condition, with peripheral saturation 95% in room-air and mild exertional dyspnea. He completed 21 days of CTX and steroid therapy and was discharged 30 days after the second admission, with an HRCT showing improvement in his parenchymal lesions (Figure 1H)**.**

## 4. Discussion

To the best of our knowledge, this is the largest case series on PJP in patients with COVID-19 pneumonia in non-HIV patients. With the exception of Patient 4, who experienced an atypical course of COVID-19, all the patients developed PJP after clinical resolution of COVID-19 pneumonia. In two out of five cases, we could not establish a proven PJP diagnosis, but only a possible diagnosis, according to EORTC/MSGERC criteria, since we could not demonstrate other microbiological evidence of *P. jirovecii* [5]. Nonetheless, in both cases, we decided to empirically treat the patients for PJP and we derived an *ex-juvantibus* diagnosis, because of clinical and radiological improvement after therapy for PJP. 

Surprisingly, the patients with proven PJP have never had BDG detectable on repeated serum samples; we explained these findings with the fact that, although serum BDG has a high negative predictive value for PJP diagnosis, its role is mostly recognized in HIV patients and its sensitivity could be lower in immunocompetent hosts [8].

Notably, all but one patient had no pre-existing immunosuppressive conditions or other risk factors for PJP development upon COVID-19 diagnosis; nonetheless, following infection with COVID-19, all the patients fulfilled at least one host factor for PJP, according to the EORTC/MSGERC consensus [5].

All the patients received at least 2 weeks of high-dose steroids. This may have been the most probable risk factor for PJP development in our cohort, together with CD4+ lymphopenia, which has been largely observed in patients affected by COVID-19, correlating with a poor prognosis, especially in younger patients [9,10].

## 5. Conclusions

The use of corticosteroids for COVID-19 respiratory impairment seems to be the most common risk factor for PJP, together with viral-induced and iatrogenic lymphopenia. The worsening in respiratory function and the characteristic radiological picture during or after COVID-19 pneumonia should raise the suspicion of PJP, even in immunocompetent patients with negative BDG assays, and should lead clinicians to actively search for *P. jirovecii* by obtaining histologic or BALF samples.

The importance of this real-life experience lies primarily in two unmet needs in COVID19 management: (1) the usefulness of CTX prophylaxis; (2) the suspicion of PJP in patients with a worsening respiratory condition who have recently recovered from COVID-19 pneumonia. With respect to CTX prophylaxis, it is well established that it is mandatory in patients being treated with a high steroid dosage (at least 25 mg prednisone equivalent administration for more than 4 weeks). Notably, in our case series, even a lower cumulative dosage of steroids resulted in PJP development. Therefore, we can speculate that in patients affected by COVID-19, CTX prophylaxis could be recommended if at least one host risk factor for PJP development is fulfilled, but further prospective studies are needed.

Finally, our series demonstrates that a precise microbiological diagnosis on and adequate specimen (BALF) is needed to optimally manage patients with COVID-19 who experience a “relapse pattern” after the first peak of the disease. Further studies are required to better establish risk factors and predictors of PJP development in COVID-19 patients and to plan the best preventive strategy. 

## Figures and Tables

**Table 1:** Patients’ characteristics. ICU = Intensive Care Unit; NHL = Non-Hodgkin lymphoma; PJP = Pneumocystis jirovecii Pneumonia.

	Patient 1	Patient 2	Patient 3	Patient 4	Patient 5	Median (IQR)
ICU	No	Yes	No	No	No	
High-flow ventilation	Yes	Yes	No	No	No	
PJP diagnosis	Proven	Possible	Proven	Proven	Possible	
Days from COVID onset	40	39	120	45	26	40 (32.5–82.5)
SARS-CoV-2 RNA at PJP diagnosis	negative	negative	positive	positive	negative	
Cumulative steroid dosage (prednisone equivalent)	962 mg	1150 mg	630 mg	400 mg	475 mg	630 (437–1056)
Total Steroid days	32	25	15	15	20	20 (15–28.5)
CD4 count at PJP diagnosis (cell/mm^3^)	895	141	93	62	1012	141 (77–953.5)
Absolute lymphocyte count at PJP diagnosis (cell/mm^3^)	1260	590	240	260	2200	590 (250–1730)
Pre-existing PJP risk factors	None	None	Rituximab NHL	None	None	
Host factors for PJP development according to EORTC/MSGERC consensus (see text)	Steroids	Steroids CD4+ count	Steroids CD4+ count Medications	Steroids CD4+ count	Steroids	

## Data Availability

Not applicable.
